# Efficacy and safety of pegzilarginase in patients below 2 years of age with arginase 1 deficiency: a phase 3, open-label, multi-centre study

**DOI:** 10.1016/j.eclinm.2026.104021

**Published:** 2026-06-29

**Authors:** Arunabha Ghosh, Anna Baghdasaryan, Patricia Lipari Pinto, Mattias Rudebeck

**Affiliations:** a Willink Biochemical Genetics Unit, Manchester University NHS Foundation Trust, Manchester, United Kingdom; b School of Biological Sciences, University of Manchester, Manchester, United Kingdom; c Paediatric Metabolic Medicine, St Luke’s Hospital, Bradford Teaching Hospitals NHS Foundation Trust, Bradford, United Kingdom; d Division of General Pediatrics, Department of Pediatrics and Adolescent Medicine, Medical University of Graz, Graz, Austria; e Unidade Local de Saúde Santa Maria, Lisboa, Portugal; f Immedica Pharma AB, Stockholm, Sweden

**Keywords:** Arginine, Arginase 1, ARG1-D, Enzyme therapy, Gross motor function measure, Hyperargininaemia, Inherited metabolic disorder, Inborn error of metabolism, Novel therapeutics, Paediatric, Rare disease, Spasticity, Urea cycle disorder

## Abstract

**Background:**

Arginase 1 Deficiency (ARG1-D) is a rare metabolic disorder characterized by marked hyperargininaemia and progressive neurological impairment. Dietary protein restriction alone is often insufficient to normalize plasma arginine (pArg) or prevent disease progression. Pegzilarginase, a recombinant human ARG1 enzyme therapy, has demonstrated pArg normalization and improved clinical outcomes in patients ≥2 years. This study evaluated safety, pharmacokinetics (PK), and activity of pegzilarginase in patients <2 years.

**Methods:**

In this phase 3, open-label, single-arm study (NCT06582524, EU CT 2024-510797-25), conducted in United Kingdom, Austria and Portugal, patients received once-weekly subcutaneous pegzilarginase. As primary endpoint, change in pArg at 12 weeks was assessed. Secondary endpoints included safety, PK, plasma ornithine and Gross Motor Function Measure (GMFM-66). Descriptive analyses were performed on the Full Analysis Set.

**Findings:**

Three patients (mean age 20.3 months) were included between 30 August 2024 and 17 June 2025. After 12 weeks, mean (SD) pArg decreased by 221.5 (81.3) μmol/L, a 70.6% (4.3) reduction, reaching normal range by Visit 4 (114.1 [68.4] μmol/L). Plasma ornithine increased and plasma ammonia remained largely normal. GMFM-66 total scores improved by 21.0 (13.9) points (18.1% [5.8%]). Pegzilarginase exposure was consistent with previous studies; AUC and half-life were similar, whereas clearance and volume of distribution were lower, as expected in infants. No new safety findings were observed.

**Interpretation:**

In patients with ARG1-D <2 years of age, pegzilarginase demonstrated pharmacokinetic and pharmacodynamic responses comparable to older children and a favourable safety profile.

**Funding:**

Immedica Pharma AB.

**Research in context**

**Evidence before this study**

We searched PubMed from database inception to Jan 31, 2025, without language restrictions, and reviewed reference lists of relevant articles. Search terms included “arginase 1 deficiency”, “ARG1-D”, “hyperargininaemia”, and “pegzilarginase”.

Available evidence is limited due to the ultra-rare nature of arginase 1 deficiency (ARG1-D) and consists mainly of small observational cohorts and a few prospective clinical studies in patients aged 2 years and older. These studies show that elevated arginine is associated with progressive neurological impairment and that dietary management alone is insufficient to achieve sustained metabolic control. Clinical trials of pegzilarginase in patients aged ≥2 years demonstrate substantial and sustained reductions in plasma arginine, with associated functional improvements and a favourable safety profile. However, no interventional studies have evaluated pegzilarginase in patients younger than 2 years of age.

**Added value of this study**

This study provides the first prospective clinical data on pegzilarginase in patients with ARG1-D younger than 2 years of age. We show that pharmacokinetic exposure, biochemical response, and short-term safety in this population are consistent with findings in older patients. Treatment rapidly normalized plasma arginine, increased ornithine concentrations, and maintained metabolic stability without inducing hyperammonaemia. The study also demonstrates that subcutaneous administration without prior intravenous exposure is feasible and well tolerated.

**Implications of all the available evidence**

Together with existing evidence, these findings support early targeting of arginine as a key driver of disease in ARG1-D and suggest that treatment initiation in infancy is feasible and may be beneficial. The availability of an effective therapy in early life strengthens the rationale for early diagnosis, including through newborn screening. Future studies should assess long-term clinical outcomes, including neurodevelopment and disease modification, following early treatment initiation.

## Introduction

Arginase 1 deficiency (ARG1-D) (ORPHA90; ICD-10 code: E72.2; OMIM: 207800) is a rare autosomal recessive urea cycle disorder characterized by marked hyperargininaemia and progressive neurological impairment.^1^, ^2^, ^3^, ^4^

ARG1-D is one of the least common urea cycle disorders (UCDs) and is an ultra-rare disease with a prevalence estimated to 1:726,000.^5^ ARG1-D is currently estimated to account for approximately 3.5% of all UCD cases.^6^

The disease results from severely decreased or non-existent arginase 1 (ARG1, EC 3.5.3.1) activity, the final enzyme of the urea cycle, which catalyses the hydrolysis of arginine into ornithine and urea. Patients thereby show accumulation of unmetabolized arginine and its metabolites, including guanidino compounds.^7^ Moreover, defective ARG1 enzymatic activity results in reduced formation of its downstream metabolite ornithine, causing mitochondrial ornithine depletion.^8^

Persistently elevated arginine and its metabolites have been reported to be the proximal or direct driver of disease manifestations and progression.^4^^,^^7^^,^^9^^,^^10^

Neuromotor complications are a hallmark of ARG1-D with lower-limb spasticity typically emerging as one of the first symptoms in early childhood. The progressive nature of the disease impairs mobility and balance, causing difficulties in walking and stair climbing, and ultimately leading to spastic diplegia, motor disability and increased morbidity.^11^, ^12^, ^13^ Beyond motor impairment, the course of ARG1-D leads to significant developmental delay, progressive cognitive decline and seizures.

Unlike other UCDs, typically presenting in the first days of life, most ARG1-D patients are asymptomatic at birth and early infancy. Typically, patients develop initial symptoms at 2–3 years of age. The clinical picture is ultimately strikingly uniform, although variation in timeframe and progression of symptoms has been observed between patients.^4^^,^^7^^,^^10^ Symptomatic hyperammonaemia and hyperammonaemic crises are relatively uncommon in ARG1-D, likely reflecting preserved upstream urea cycle function that permits continued ammonia detoxification despite arginase 1 deficiency.^9^ However, severe episodic hyperammonaemia can still occur, particularly during febrile illness.

Diagnosis can be readily made with assessment of plasma/serum arginine concentrations, determination of arginase activity in red blood cells, or performing genetic analysis.^14^ Delays in diagnosis may occur due to both the overlap in symptomatology with other diseases, such as cerebral palsy or hereditary spastic paraplegia,^11^^,^^15^^,^^16^ as well as lack of disease awareness.

Long-term management of patients with ARG1-D aims to prevent disease onset and halt or reverse symptom progression as well as to prevent hyperammonaemia by reducing plasma arginine levels while ensuring conditions allowing normal growth and development.^14^

Disease management with dietary protein restriction has been shown to lower plasma arginine levels in some patients, with amelioration of some of the disease-related abnormalities, thus providing support for the therapeutic value of arginine reduction.^9^^,^^17^, ^18^, ^19^, ^20^, ^21^ However, this approach is inadequate in most patients as demonstrated by the persistence of marked hyperargininaemia and ongoing disease progression, mainly due to inability to address endogenous arginine production.^9^ Additionally, dietary management is challenging, especially in growing children, and necessitates supplementation with an unpalatable essential amino acids (EAA) formula to maintain a safe amino acid intake.^6^^,^^12^^,^^14^^,^^21^

Early targeted intervention in ARG1-D is critical. The first years of life represent a period of rapid neurodevelopment when the brain is especially vulnerable to metabolic disturbances.^4^^,^^10^ Timely biochemical correction of arginine levels may therefore mitigate disease progression and optimize long-term functional outcomes, highlighting the importance of initiating therapy before substantial clinical manifestations occur.^19^^,^^22^ Thus, novel therapeutic strategies targeting arginine, the pathogenic metabolite, ideally initiated in early infancy or childhood, before symptom onset, are needed.

Pegzilarginase is a cobalt-substituted, recombinant human ARG1 enzyme that is covalently conjugated to monomethoxy polyethylene glycol (mPEG) and metabolizes plasma arginine.

Previous clinical studies addressing impact of pegzilarginase in ARG1-D included patients aged 2 years and above and demonstrated normalization of plasma arginine accompanied by clinically relevant functional mobility improvements,^23^ with sustained biochemical effect and further clinical improvements during longer-term treatment.^24^ Based on these studies, pegzilarginase (Loargys®) was granted a marketing authorization in the EU and UK for the treatment of ARG1-D in patients aged 2 years and older in December 2023, in Oman in November 2025, in the United States in February 2026, and in Canada in May 2026.

This study was designed to evaluate the safety, pharmacokinetics, and activity of subcutaneous pegzilarginase for the treatment of ARG1-D in patients below 2 years of age.

## Methods

The study was conducted in accordance with International Council for Harmonisation (ICH) Good Clinical Practice Guidelines and applicable laws and regulations. The research protocol was approved by all relevant ethics committees for all study sites (Wales Research Ethics Committee 5 Bangor, Cardiff, United Kingdom [reference number: 24/WA/0141]; authorization in the European Union [Austria and Portugal] was performed via an integral procedure in accordance with Clinical Trials Regulation EU No 536/2014 through the Clinical Trials Information System [CTIS], including ethics approval in participating member states [EU CT No: 2024-510797-25]). All procedures were conducted in accordance with ethical standards of the responsible committee on human experimentation (institutional and national).

### Trial design and treatment

This was an open-label, single-arm, non-controlled, repeat dosing, multicentre study conducted at three sites in three countries (United Kingdom, Austria, and Portugal; clinicaltrials.gov NCT06582524, EU CT 2024-510797-25). It consisted of a screening period, a 12-week treatment period, and a safety follow-up period of eight weeks. Patients continued their current individualized disease management (protein restriction, EAA, ammonia scavengers, if prescribed) regimen determined as standard of care for the duration of the study.

Patients were initiated and continuously administered pegzilarginase (Loargys®, Immedica Pharma AB, Stockholm, Sweden) as subcutaneous injections throughout the study. The dose selection was derived using a population pharmacokinetic-pharmacodynamic (PK-PD) model developed with PK and PD data with subcutaneously administered pegzilarginase from previous studies in the pegzilarginase clinical development program, which included data from patients ≥2 years of age. Using World Health Organization (WHO) weight for age tables, a dataset of 1000 virtual paediatric patients was generated by sex and age category (0–<1 month, 1–<3 months, 3–<6 months, 6–<12 months and 12–24 months). The modelling determined that the same once weekly starting dose as older patients, 0.1 mg/kg, was expected to provide adequate control of arginine prior to dose titrations in a majority of the patients aged <24 months. This starting dose combined with a dose increment of 0.05 mg/kg was expected to yield optimal control of arginine levels over the dosing interval, i.e., minimizing the time that arginine remained below the lower limit of the normal and maximizing the time that arginine remained in the predefined normal range.

Dosing modifications were possible according to the protocol at Visits 5 and 9 (i.e., after four and eight doses) based on two prior plasma arginine assessments out of range 50–150 μmol/L. The volume of the subcutaneous dose was to be calculated based on the patient’s weight at Baseline, unless the weight had changed by ≥ ± 10% of the baseline weight when the new weight was to be used to calculate the dose volume.

### Patients

Key inclusion criteria were age <24 months; documented ARG1-D diagnosis (through elevated plasma arginine, pathogenic variants in *ARG1* (HGNC 663), and/or diminished red blood cell ARG1 activity); plasma arginine ≥180 μmol/L. Patients also had to weigh >8 kg due to the clinical study related blood collection volumes required. Key exclusion criteria were symptomatic hyperammonaemia (ammonia >100 μmol/L requiring acute care or hospitalization) within four weeks before first pegzilarginase dose; active infection requiring anti-infective therapy within two weeks before first pegzilarginase dose, medical conditions or comorbidities that would preclude study compliance or data interpretation; botulinum toxin use within 16 weeks prior to first pegzilarginase dose to avoid confounding effects on mobility assessments, or prior liver or hematopoietic transplant.

All patients’ parent/guardian provided written informed consent before initiation of screening procedures and therapeutic intervention.

### Assessments and outcomes

The primary efficacy endpoint was change from baseline in plasma arginine after 12 weeks of pegzilarginase treatment.

The secondary efficacy endpoints were:

• PK parameter evaluation including half-life (t_½_), time to maximum observed concentration (t_max_), maximum observed concentration (C_max_), area under the plasma drug concentration-time curve from time 0 to time t (AUC_0-t_), area under the plasma drug concentration-time curve from time 0 extrapolated to infinite time (AUC_0-∞_), extravascular clearance (CL/F), and apparent volume of distribution at steady state after non-intravenous administration (V_ss_/F). Due to the paediatric population, blood sampling was kept at a minimum. Hence, blood samples for measurement of serum concentrations of pegzilarginase were collected pre-dose and once between 24 and 48 h post-dose at PK visits,
• PD response evaluation: antidrug antibodies (ADAs) against pegzilarginase and/or polyethylene glycol (PEG), levels of plasma arginine and plasma ornithine,
• Changes in physical function after 12 weeks of pegzilarginase treatment as measured by Gross Motor Function Measure (GMFM)-66 Parts A through E, as age appropriate and feasible. The GMFM evaluates unaided gross motor function (performed without bracing or assistive devices) by observing the patient’s ability to initiate and complete certain movements. The GMFM-66 consists of 66 Items across 5 parts (A-E) capturing tasks involving lying and rolling (A), sitting (B), crawling and kneeling (C), standing (D), and walking, running, and jumping (E). GMFM-66 items are ordered in terms of difficulty. Individual tasks are scored as: 0 = does not initiate, 1 = initiates, 2 = partially completes, 3 = completes, or NT = not tested. The total score reflects the sum of all scored tasks.^25^

Safety and tolerability were evaluated through adverse events (AEs), vital signs, physical examinations, laboratory testing, growth assessments (length, weight, and head circumference) and electrocardiograms (ECGs). AEs of special interest (AESIs) included hypersensitivity reactions (HSRs) and injection site reactions (ISRs) (as potential occurrences with biologic/enzyme therapies), prolonged hypoargininaemia, and hyperammonaemic episodes (as known disease occurrences). Prolonged hypoargininaemia was defined as events of continuous plasma arginine levels below the lower limit of normal for 14 days or longer, while hyperammonaemic episodes were defined as ammonia ≥100 μmol/L with related symptoms requiring hospitalization or emergency room treatment. AEs were coded using Medical Dictionary for Regulatory Activities (MedDRA) version 27.0.

### Bioanalytical methods for the determination of plasma arginine and ornithine

Plasma arginine and ornithine concentrations were analysed at a central laboratory (Charles River Laboratories, Den Bosch, the Netherlands) using a LC-MS/MS assay. Blood samples were collected in tubes containing the arginase inhibitor Nω-Hydroxy-nor-L-arginine (nor-NOHA) to prevent *ex vivo* reduction of arginine levels with pegzilarginase.

### Sample size

Given the ultra-rare nature of ARG1-D and the additional restriction that eligible patients had to be diagnosed, identified, enrolled, and initiated on study treatment before reaching 2 years of age, the number of potentially recruitable patients was considered to be extremely limited from an operational and epidemiological perspective. The sample size of three patients was solely based on the rarity of the disease, clinical considerations and the regulatory authority agreed plan.

### Data handling

An electronic data capture system (Viedoc™, Uppsala, Sweden) was used for the collection of clinical data at all investigational sites. Laboratory data was held within individual laboratories’ databases, and all data was merged into the data analysis database held by FGK Clinical Research GmbH (Munich, Germany).

### Statistical analysis

The full analysis set (FAS) was used for the analysis of all study endpoints. The FAS consisted of all patients who were enrolled and received at least one dose of pegzilarginase.

All study endpoints were analysed descriptively. No inferential tests were planned. For qualitative variables, the number and percentage in each category were calculated. For quantitative variables, the mean, standard deviation, minimum, median, and maximum were provided. All analyses were done on a valid case basis, i.e., no imputation techniques were applied for missing observations.

Although statistical testing was not prespecified, post-hoc analyses of paired data were performed to estimate 95% CIs for percentage change from Baseline using Student’s t-test. Given the small sample size, these analyses should be interpreted as exploratory and indicative of the magnitude of change in clinical outcomes.

Statistical programming and analyses were performed using SAS® Version 9.4.

### Role of the funding source

The funder of the study was involved throughout the study, including study design, data collection, data analysis, data interpretation, writing of the report and manuscript.

## Results

### Patients

**Fig. 1:**
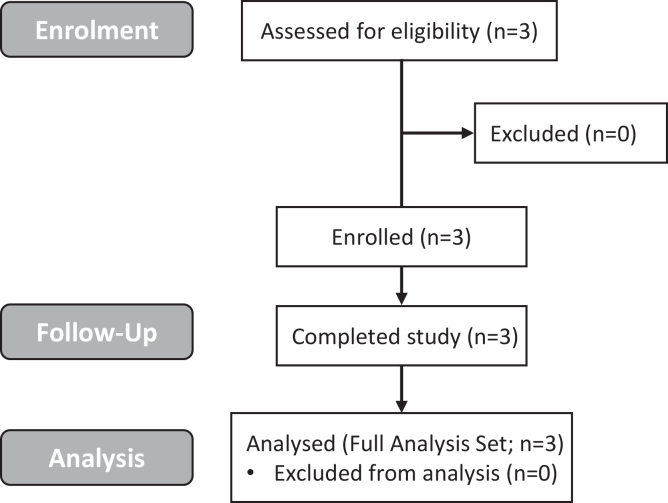
**Patient flow.** The target enrolment was achieved, and all patients completed the study.

From 30 August 2024 to 14 January 2025, three patients were screened, met study criteria and were enrolled. All patients completed the study (Fig. 1).

Two male patients (66.7%) and one female patient (33.3%) were included in the study. Mean (standard deviation [SD]) age at enrolment was 20.3 (4.6) months (range: 15–23 months). All patients were managed with dietary protein restriction. One patient was treated with an ammonia scavenger, glycerol phenylbutyrate. The treatment was initiated before study start and continued throughout the study. Despite standard-of-care treatment, mean (SD) pArg was markedly elevated; 312.0 (107.53) μmol/L.

All patients were diagnosed early, two by newborn screening (sibling being an index patient). The two patients who underwent DNA testing had both homozygous variants, one with c.295G>A (p. Gly99Arg), and one with c.32T>A (p.Ile8Lys). Observed ARG1-D clinical symptoms were reported in two of the three patients at enrolment (Table 1). One patient had increased alanine aminotransaminase (ALT) and aspartate aminotransaminase (AST) values as well as an increased prothrombin time/international normalized ratio. The other patient presented with elevated ALT and AST values as well as spasticity and language delays. Baseline motor function (walking and climbing) was normal in two patients while one patient was not able to walk or climb stairs. None of the patients used assisted devices.

**Table 1:** Key demographics, clinical characteristics, and baseline assessments (full analysis set).

Patient information	Pegzilarginase (N = 3)
Age at enrollment, months	
Mean (SD)	20.3 (4.6)
Median (Q1, Q3)	23.0 (15.0, 23.0)
Min, max	15, 23
Sex, n (%)	
Male	2 (66.7)
Female	1 (33.3)
Race, n (%)	
White/Caucasian	2 (66.7)
Black or African American	1 (33.3)
Weight (kg)	
Mean (SD)	10.4 (1.9)
Median (Q1, Q3)	9.8 (8.8, 12.5)
Min, max	8.8, 12.5
Height (cm)	
Mean (SD)	81.5 (4.2)
Median (Q1, Q3)	81.0 (77.5, 85.9)
Min, max	77.5, 85.9
Key medical history, n (%)	
Spasticity	
Any	1 (33.3)
Lower-limb	1 (33.3)
Upper limb	0
Seizures	0
Liver test abnormalities	2 (66.7)
Hyperammonaemia	0
Cognitive delays	0
Language delays	1 (33.3)

Max, maximum; min, minimum; n/N, number; Q, quartile; SD; standard deviation.

### Efficacy

**Fig. 2:**
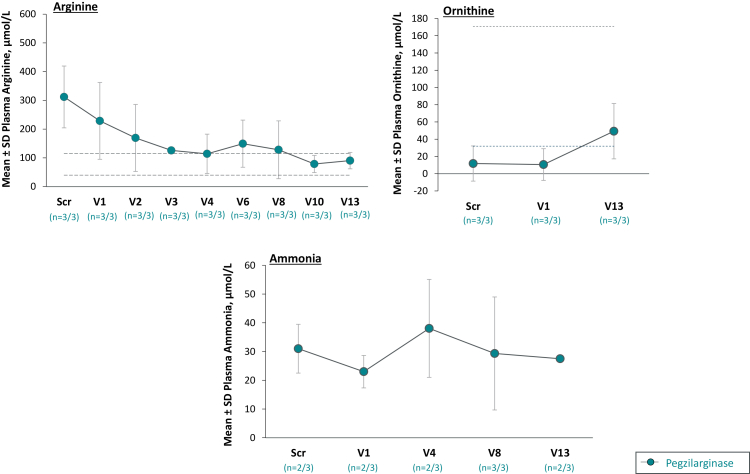
**Effect of pegzilarginase on biochemical endpoints (full analysis set).** Normal range for plasma arginine is 40–115 μmol/L.^4^ Normal range for plasma ornithine is 32–171 μmol/L.^26^ In two patients, plasma ornithine levels were below the lower limit of quantification at Screening and Visit 1 and set to ‘0’. Scr, screening; V, visit.

The mean (SD) absolute change from Baseline in plasma arginine after 12 weeks of pegzilarginase treatment was −221.53 (81.26) μmol/L corresponding to a mean (SD) percentage reduction from Baseline of 70.63% (4.30) (Table 2; Fig. 2).

**Table 2:** Clinical outcomes (full analysis set).

Endpoint	Baseline	Week 12	Percentage change from baseline
Primary			
Arginine, plasma, μmol/L			
N	3	3	3
Mean (SD)	312.0 (107.53)	90.5 (28.5)	−70.6 (4.3)
Median (Q1, Q3)	271.0 (231.0, 434.0)	78.7 (69.7, 123.0)	−71.7 (−74.3, −65.9)
Min, Max	231.0, 434.0	69.7, 123.0	−74.3, −65.9
95% CI^h^	–	–	−81.3, −60.0
Secondary			
GMFM-66, points			
N	3	3	3
Total score, mean (SD)^a^	105.7 (55.8)	126.7 (69.6)	+18.1 (5.8)
95% CI^h^	–	–	3.8, 32.3
GMFM-A, mean (SD)^b^	12.0 (0)	12.0 (0)	0 (0)
95% CI^h^	–	–	–
GMFM-B, mean (SD)^c^	35.3 (7.0)	38.0 (6.9)	+7.9 (8.4)
95% CI^h^	–	–	−12.9, 28.8
GMFM-C, mean (SD)^d^	17.0 (13.7)	20.7 (13.7)	+62.8 (77.2)
95% CI^h^	–	–	−128.9, 254.5
GMFM-D, mean (SD)^e^	16.3 (13.6)	21.7 (17.9)	+23.3 (23.8)
95% CI^h^	–	–	−35.9, 82.4
GMFM-E, mean (SD)^f^	25.0 (23.3)	34.3 (31.9)	+37.5 (0.6)
95% CI^h^	–	–	31.7, 43.2
Ornithine, plasma, μmol/L^g^			
N	3	3	1
Mean (SD)	11.8 (20.4)	49.3 (32.1)	+143.8 (−)

CI, confidence interval; GMFM, Gross Motor Function Measure, with dimensions A-E; Q, quartile; SD, standard deviation.

a Possible score range, 0–198 points; lower scores indicate greater functional mobility impairment.

b Possible score range, 0–12 points; lower scores indicate greater functional mobility impairment.

c Possible score range, 0–45 points; lower scores indicate greater functional mobility impairment.

d Possible score range, 0–30 points; lower scores indicate greater functional mobility impairment.

e Possible score range, 0–39 points; lower scores indicate greater functional mobility impairment.

f Possible score range, 0–72 points; lower scores indicate greater functional mobility impairment.

g Normal range for ornithine 32–171 μmol/L.^26^ In two patients, plasma ornithine levels were below the lower limit of quantification at Baseline and set to ‘0’ for mean change calculation.

h Post-hoc exploratory analyses using Student’s t-test.

By Visit 4, mean plasma arginine had reached normal range (114.13 μmol/L; SD 68.36 μmol/L). One patient required a dose increase at Visit 9; at the following visits (Visits 10 and 13), mean population plasma arginine values were within the normal range. At the final treatment visit (Visit 13), the patient had a plasma arginine concentration of 123 μmol/L, which was close to the upper limit of normal of 115 μmol/L (as defined in the study).

Plasma ornithine levels were below the lower limit of quantification at Screening and Visit 1 in two patients. In these, the ornithine level at Visit 13 was 30.5 μmol/L and 31.0 μmol/L, respectively (normal reference range: 32–171 μmol/L^26^). In one patient, the plasma ornithine level was 35.4 μmol/L at Screening and 86.3 μmol/L at Visit 13 (Table 2; Fig. 2).

Plasma ammonia values were normal at Screening in all patients and remained normal in two of the three patients. One patient had slightly increased ammonia values at Visits 4, 8, and the final safety follow-up visit (Fig. 2). All abnormal plasma ammonia results were considered not clinically significant by the investigator.

For changes in motor function, the GMFM-66 total score (dimensions A-E) increased from Baseline by a mean (SD) of 21.0 (13.9) points, corresponding to a mean (SD) increase of 18.1% (5.75). No change was observed in dimension A (lying and rolling). In dimensions B (sitting), C (crawling and kneeling), D (standing), and E (walking, running and jumping), the mean (SD) improvements from Baseline were 2.7 ± 3.1, 3.7 ± 3.1, 5.3 ± 5.0, and 9.3 ± 8.6 points, respectively (Table 2).

In one patient, a clinically significant abnormal increased tone in both lower limbs and spasticity, hyperreflexia and bilateral clonus were reported at Screening, which were linked to the patient’s medical history of muscle spasticity and dystonia. The symptoms persisted during the study but during treatment they were assessed as not clinically significant by the investigator, whereas a noticeable worsening was observed at the final safety follow-up visit, seven weeks following completion of pegzilarginase treatment, which the investigator considered clinically significant.

### Pharmacokinetics

**Fig. 3:**
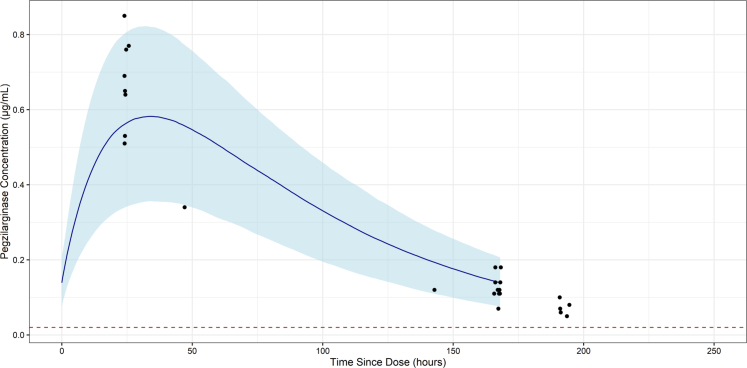
**Plasma pegzilarginase concentrations in patients <2 years compared to concentrations in older patients (full analysis set).** Observed pegzilarginase concentrations in patients below 2 years of age were within the prediction interval of the prior model developed in paediatric and adult population above 2 years of age. Points = observed pegzilarginase concentrations, blue line = simulated pegzilarginase concentrations in patients >2 years of age, blue shaded area = 95% prediction interval, horizontal dashed red line = Lower limit of quantification (0.02 μg/mL).

The observed pegzilarginase concentrations in these patients <2 years of age were generally within the prediction interval of the model developed in previous studies in patients ≥2 years of age (Fig. 3).

Pharmacokinetic parameters derived from pegzilarginase serum concentration data are provided in Table 3. Following doses of 0.1 mg/kg, the patients had exposures comparable to those estimated in patients ≥2 years of age. AUC_0-168_ and t_1/2_ were approximately similar between patients aged ≥2 years and this population <2 years. However, clearance (CL) and volume of distribution were lower in the population <2 years of age, although this was expected due to the allometrically scaled parameters. In the <2 years group, the geometric mean (95% CI) CL/F was 18.9 (9.5, 37.7), and V_ss_/F was 1190 (623, 2289), although the precision of these estimates is limited by the small sample size. The bioavailability in the population <2 years of age was slightly higher than in the older population.

**Table 3:** Pharmacokinetic variables (Full Analysis Set).

Occasion	Dose (mg)	C_max_ (μg/mL)	t_max_ (h)	AUC_0–168h_ (h × μg/mL)	AUC_0-∞_ (h × μg/mL)	CL (mL/h)	V_c_ (mL)	V_p_ (mL)	V_ss_ (mL)	F (−)	CL/F (mL/h)	V_c_/F (mL)	V_p_/F (mL)	V_ss_/F (mL)	t_1/2_ (h)
First															
GeoMean	1.03	0.470	33.3	45.9	54.2	12.1	567	198	765	64.1	18.9	885	309	1190	51.9
GeoMean CV%	18.1	14.4	6.34	7.86	10.6	18.1	18.1	12.7	16.8	9.60	28.3	28.2	22.6	26.7	1.16
Min	0.880	0.422	31.0	43.1	50.3	10.2	478	178	655	58.6	14.3	673	250	924	51.3
Median	0.980	0.446	34.0	44.7	51.7	12.0	558	192	750	63.4	19.0	881	303	1180	51.8
Max	1.25	0.553	35.0	50.1	61.1	14.6	684	228	912	70.9	24.9	1170	390	1560	52.5
Last															
GeoMean	1.22	0.646	31.0	64.6	74.5	12.1	567	198	765	64.1	18.9	885	309	1190	52.0
GeoMean CV%	25.9	24.6	5.69	23.4	23.1	18.1	18.1	12.7	16.8	9.60	28.3	28.2	22.6	26.7	0.836
Min	0.930	0.499	29.0	51.3	59.4	10.2	478	178	655	58.6	14.3	673	250	924	51.7
Median	1.27	0.668	32.0	64.7	74.2	12.0	558	192	750	63.4	19.0	881	303	1180	51.8
Max	1.54	0.808	32.0	81.4	93.7	14.6	684	228	912	70.9	24.9	1170	390	1560	52.5
≥2 years of age^a^															
GeoMean	3.10	0.579	34.0	61.3	–	29.6	1350	447	–	56.9	–	–	–	–	50.2
GeoMean CV%	–	19.9	–	18.3	–	42.3	41.6	38.3		21.2	–	–	–		7.53

Note: Data from all three patients were included for the calculation of summary statistics.

AUC_0–168h_ = area under the plasma drug concentration-time curve from 0 to 168 h, AUC_0-∞_ = area under the plasma drug concentration-time curve from 0 to infinity, CL = clearance, CL/F = apparent clearance, C_max_ = maximum observed concentration, CV = coefficient of variation, F = bioavailability, GeoMean = geometric mean, Max = maximum, Min = minimum, t_1/2_ = half-life, t_max_ = time to maximum concentration, V_c/p_ = central/peripheral volume of distribution, V_c/p_/F = apparent volume of distribution for the central/peripheral compartment, V_ss_ = volume of distribution at steady state, V_ss_/F = apparent volume of distribution at steady state (V_c_/F + V_p_/F, where V_c_/F and V_p_/F are empirical Bayes estimates from the population pharmacokinetic model).

a Data for patients aged ≥2 years from prior analysis.

### Exposure, safety, and tolerability

#### Treatment exposure

All three patients received all weekly doses according to the study protocol. In one patient, the dose was increased from 0.1 mg/kg to 0.15 mg/kg at Visit 9, while the other two patients received 0.1 mg/kg/week throughout the study.

#### Adverse events

In this study, no major safety signals were observed for pegzilarginase initiated subcutaneously. No serious AEs or deaths were reported. All reported treatment-emergent AEs (TEAEs) were considered mild in severity, and all but one (Injection site erythema) were assessed as unrelated to pegzilarginase administration by the investigator.

All patients experienced at least one TEAE from the MedDRA system organ class (SOC) ‘General disorders and administration site condition’ and at least one TEAE from the SOC ‘Infections and infestations’. Half of the TEAEs were classified within these two SOCs (Table 4). Except for the TEAE Pyrexia, which was observed in two patients (67%), all TEAEs were observed in a single patient only (33%).

**Table 4:** Summary of key safety observations (full analysis set).

Exposure and AEs	Pegzilarginase (N = 3)
Exposure and compliance	
Treatment exposure, weeks	12
Dosing compliance, %	100
Summary of AEs, n (%)	
Any treatment-emergent AE (TEAE)	3 (100.0)
Mild	3 (100.0)
Moderate	0
Severe	0
TEAE leading to discontinuation	0
TEAE leading to dose interruption	0
TEAE leading to death	0
Serious AEs	0
Other significant AEs	
Hypersensitivity reaction	0 (0)
Injection site reaction	1 (33.3)
Hyperammonaemic events^a^	0
Prolonged hypoargininaemia^b^	0
TEAEs with ≥15% incidence, n (%)^c^	
Anemia	1 (33.3)
Coagulation time prolonged^d^	1 (33.3)
Cough	1 (33.3)
Condition aggravated^d^	1 (33.3)
Diarrhea	1 (33.3)
Febrile infection	1 (33.3)
Injection site erythema	1 (33.3)
Muscle spasticity^d^	1 (33.3)
Productive cough	1 (33.3)
Pyrexia	2 (66.7)
Respiratory syncytial virus infection	1 (33.3)
Respiratory tract infection	1 (33.3)
Respiratory tract infection bacterial	1 (33.3)
Rhinorrhea	1 (33.3)
Upper respiratory tract infection	1 (33.3)
Vomiting	1 (33.3)

AE, adverse event; n/N, number; TEAE, treatment-emergent AE.

a Defined as events that met all of the following criteria: (1) hyperammonaemia with a confirmed ammonia level ≥100 μM, (2) symptoms related to hyperammonaemia, (3) requiring hospitalization or emergency room management, with or without admission to the hospital.

b Defined as events of continuous plasma arginine levels below the lower limit of normal for 14 days or longer.

c Due to sample size, includes all.

d At safety follow-up seven weeks after completion of pegzilarginase treatment.

No clinically significant abnormalities were reported in clinical chemistry parameters.

All three patients had at least one abnormal haematology value reported during the study, where the majority were considered not clinically significant by the investigators. Clinically significant abnormalities in red blood cells, haemoglobin, and haematocrit were observed in one patient at the final safety follow-up visit after completion of pegzilarginase treatment. The abnormalities were reported as a TEAE of Anaemia, which was reported as resolved.

Except for a clinically significant abnormal prothrombin time observed in one patient at the final safety follow-up visit after completion of pegzilarginase treatment, no clinically significant abnormalities in coagulation parameters were observed. No intervention was required, and the investigator assessed these to be a sign of ARG1-D where similar abnormalities have been reported previously.^27^

No AE led to dose interruption or pegzilarginase withdrawal. There were no hypersensitivity reactions, hyperammonaemic events or prolonged hypoargininaemia reported. Only a single injection site reaction (injection site erythema) was reported which resolved with uninterrupted treatment. No clinically meaningful adverse changes from baseline in growth parameters were observed during the study.

#### Anti-drug antibodies

No ADAs were detected against either the PEG component or pegzilarginase in any patient.

## Discussion

This study provides the first systematic evaluation of the pharmacokinetics, pharmacodynamics, and safety of pegzilarginase in patients younger than 2 years of age with ARG1-D. Despite inherent challenges of investigating an ultra-rare disorder in a young population, the findings provide supportive evidence that pegzilarginase exposure, biochemical activity, and short-term safety in this age group are consistent with observations previously reported in older patients.

Pharmacokinetic analyses showed that systemic exposure to pegzilarginase in patients below 2 years of age fell within the predicted range derived from prior studies in patients aged 2 years and older. Key exposure parameters, including AUC and terminal half-life, were broadly comparable across age groups, while differences in clearance and volume of distribution were consistent with expected allometric scaling in infancy. These results indicate that weight-based dosing adequately accounts for developmental physiological differences and that no clinically meaningful alterations in drug disposition were observed in this young population.

Consistent with the observed pharmacokinetic profile, pegzilarginase treatment resulted in marked clinically meaningful reductions in plasma arginine concentrations. Mean arginine levels were reduced to the predefined normal range within the first month of treatment and remained largely controlled throughout the study period, an outcome rarely seen with previous standard of care in ARG1-D due to inability to address endogenous arginine production. The magnitude of arginine lowering was comparable to that reported in older patients receiving pegzilarginase, supporting the biological plausibility and translational relevance of treatment in patients below 2 years of age. Given that persistently elevated arginine is a key driver of disease pathology in ARG1-D, achieving biochemical control during early life—a period of rapid neurodevelopment and significant vulnerability to the consequences of metabolic imbalance—may be particularly relevant. Persistent elevated plasma arginine has been shown to correlate with consistent and progressive debilitating neurologic and functional impairments, whereas reductions in plasma arginine improved manifestations.^9^

In addition, pegzilarginase increased plasma concentrations of the downstream metabolite ornithine, indicating restoration of urea cycle flux. Plasma ammonia levels remained largely within normal ranges, and no hyperammonaemic episodes were observed, further supporting the biochemical safety of pegzilarginase in this population. Of note, at least one patient had experienced episodes of significant metabolic derangement during febrile infections prior to enrolment. Despite febrile infections being observed in patients during the therapeutic intervention, no metabolic derangement and no episode of hyperammonaemia was observed, which underlines the beneficial impact of targeted therapy in this susceptible age group. However, the impact of intracellular metabolic changes and the long-term clinical implications of early intervention require further investigations.

Guanidino compounds, which are considered important pathogenic candidates in ARG1-D driven by elevated arginine, were not evaluated in the current study. However, previous pegzilarginase studies in older patients demonstrated that reductions in plasma arginine were accompanied by the same general patterns of reductions in guanidino compound concentrations,^23^^,^^24^ supporting the mechanistic relationship between arginine lowering and downstream metabolic improvement.

For motor function outcomes, one patient presented with disease-specific symptoms prior to the study enrolment with no worsening during pegzilarginase treatment. Upon treatment discontinuation, the patient experienced clinically relevant deterioration as reported at the final study visit, seven weeks after completion of study treatment. No other patient presented with disease-specific symptoms at enrolment and showed no deterioration during pegzilarginase treatment. It should be considered that the patients were young and that the treatment period was only 12 weeks in this slowly progressive disease.

No major safety signals were observed for pegzilarginase in this study population. No serious adverse events, hypersensitivity reactions, hyperammonaemic episodes, or prolonged hypoargininaemia were reported, and no patients developed anti-drug antibodies against either pegzilarginase or the PEG component. The safety profile observed in this study was consistent with previously reported findings,^23^^,^^24^ and no new or unexpected safety signals emerged. It is also important to notice that this study is the first to initiate pegzilarginase treatment using the subcutaneous route without prior intravenous dosing, and this approach was associated with acceptable tolerability and preserved pharmacodynamic activity.

As already highlighted, early detection and initiation of targeted therapy is critical for preventing the neurodevelopmental injury and improving the long-term outcome in affected patients. In this context, the increasing implementation of newborn screening programs proves essential for identification of presymptomatic patients. Furthermore, data on therapies with a well-characterised biochemical and clinical effect alongside an acceptable safety profile in early life are particularly relevant. With the availability of pegzilarginase, ARG1-D may be considered to meet several key Wilson–Jungner criteria for newborn screening,^28^ including disease severity, detectability in the pre-symptomatic period, and the presence of an intervention with potential benefit when initiated early. Jurisdiction-specific assessments can facilitate the integration of ARG1-D into newborn screening programmes in countries where it is not already included and further elucidate the benefits of early detection for long-term clinical outcomes.

Several limitations should be acknowledged. The small sample size reflects the rarity of ARG1-D and the difficulty of enrolling patients under 2 years of age limits the ability to detect uncommon adverse events or subtle pharmacokinetic differences. Due to the clinical study related blood collection volumes required, ethics committees also required patients to weigh >8 kg, further limiting the data in the youngest patients. Given that only a few patients are expected to be born with the disease annually in Europe, the number of patients enrolled in this study is considerable. In addition, the relatively short duration of treatment precludes conclusions regarding long-term safety, neurodevelopmental outcomes, or disease modification. Future data with extended follow-up will be necessary to better characterize the clinical impact of early pegzilarginase treatment. Improvements in motor function should be interpreted cautiously given the open-label design with no blinded assessors and also the potential contribution of normal developmental maturation in this age group with ARG1-D should be considered.

In conclusion, this study provides supportive evidence that patients with ARG1-D below 2 years of age exhibit pharmacokinetic and pharmacodynamic responses to pegzilarginase comparable to those of older patients, with no new safety findings. Collectively, these results indicate an important rationale for early therapeutic intervention in ARG1-D.

## Contributors

Arunabha Ghosh was the International Coordinating Investigator and contributed to data collection, data interpretation, data verification, and review of the manuscript.

Anna Baghdasaryan and Patricia Lipari Pinto contributed to data collection, data interpretation, data verification, and review of the manuscript.

Mattias Rudebeck contributed to the literature search, study design, data analysis, data interpretation, wrote the manuscript, and accessed and verified the data.

All authors approved the final manuscript. All authors had full access to data in the study and had final responsibility for the decision to submit for publication.

## Data sharing statement

Individual data generated in this study are included in this published article. Given the small sample size and the presentation of individual patient data, no additional data are available to protect patient confidentiality. Joint research proposals will be assessed based on the scientific merit of the proposal (i.e., the proposal should be scientifically sound, ethical, and have the potential to contribute to the advancement of public health) and the feasibility of the collaborative research proposal (i.e., the requesting research team must be scientifically qualified and have the resources for the proposed project). Furthermore, the study protocol and statistical analysis plan can be made available at this time. Proposals should be directed to the study sponsor. Data requestors will need to sign a data access agreement.

## Declaration of interests

Arunabha Ghosh has received consulting fees from Immedica Pharma, Fiecon Ltd and travel grants from Sanofi.

Anna Baghdasaryan has received travel grants, speaker and consulting fees from Immedica Pharma in relation to ARG1-D.

Patrícia Lipari Pinto declares no conflicts of interest.

Mattias Rudebeck is employee of Immedica Pharma AB and owns stocks or equity in the company and in Spyre Therapeutics, Inc. (formerly Aeglea BioTherapeutics, Inc.).

Immedica Pharma AB funded the study.
